# Management of biliary diseases after the failure of initial needle knife precut sphincterotomy for biliary cannulation

**DOI:** 10.1038/s41598-021-94361-8

**Published:** 2021-07-22

**Authors:** Min-Hao Lo, Cheng-Hui Lin, Chi-Huan Wu, Yung-Kuan Tsou, Mu-Hsien Lee, Kai-Feng Sung, Nai-Jen Liu

**Affiliations:** Department of Gastroenterology and Hepatology, Chang Gung Memorial Hospital and Chang Gung University College of Medicine, 5 Fu-Shin Street, Kweishan, Taoyuan 333 Taiwan

**Keywords:** Gastroenterology, Gastrointestinal diseases

## Abstract

Endoscopic retrograde cholangiopancreatography is not always successful even with needle knife precut sphincterotomy (NKPS). How to manage these patients with initial NKPS failure has not been well studied. We report the outcomes of patients who received endoscopic and non-endoscopic rescue treatment after the initial NKPS failure. During the 15 years from 2004 to 2018, 87 patients with initial NKPS failure received interval endoscopic treatment (IET group, n = 43), percutaneous transhepatic biliary drainage (PTBD group, n = 25), or bile duct surgery (BDS group, n = 19) were retrospectively studied. Compared with the PTBD group, the prevalence of choledocholithiasis was higher (69.8% vs. 16.0%, p < 0.001), and malignant bile duct stricture were lower (20.9% vs. 76.0%, p < 0.001) in the IET group. Furthermore, the IET group had a significantly longer time interval between the first and second treatment procedures (4 days vs. 2 days, p = 0.001), a lower technique success rate (79.1% vs. 100%, p = 0.021), and a shorter length of hospital stay (7 days vs. 18 days, p < 0.001). Compared to the BDS group, the only significant finding was that the patients in the IET group were older. Although not statistically significant, the complication rate was lowest in the IET group (7.0%) while highest in the BDS group (15.8%). Complications in the IET group were also mild, as compared with the other two groups. In conclusion, IET should be considered after initial failed NKPS for deep biliary cannulation before contemplating more invasive treatment such as BDS. PTBD may be the alternative therapy for patients with malignant biliary obstruction.

## Introduction

Endoscopic retrograde cholangiopancreatography (ERCP) has been widely used in the treatment of biliary diseases in recent decades. Deep bile duct cannulation is a critical step for the success of therapeutic ERCP but it cannot be always successful^1^. Conventional biliary cannulation methods include the use of a catheter or sphincterotome with or without guidewire assistance^2^_._ Using conventional methods, bile duct cannulation cannot be achieved in about 5%–15% of cases^3^. In this case, needle knife precut sphincterotomy (NKPS) is often used as a rescue technique^3–7^. However, the NKPS technique is very difficult and carries the risk of pancreatitis and perforation^8^. As a result, it was reported that the initial success rate of NKPS ranges widely from 71.3 to 92%^9–16^. Once NKPS fails, how to manage these patients optimally has not been well studied. In clinical practice, either endoscopic methods such as interval ERCP and percutaneous-transhepatic-endoscopic rendezvous procedures (PTE-RVs) or non-endoscopic methods such as percutaneous transhepatic cholangiography and drainage (PTCD) or bile duct surgery (BDS), can be the option of the rescue treatment^17–23^. However, no studies have compared the results of these rescue treatment methods in patients with initial NKPS failure. Therefore, we conducted this study to report the outcomes of patients with biliary diseases who received endoscopic and non-endoscopic rescue treatment after the initial NKPS failure due to difficult biliary cannulation.

## Patients and methods

### Definition of difficult biliary cannulation

Bile duct cannulation methods in our department included cannulation using a cannula and/or a sphincterotome, with or without guidewire assistance. After the cannula/sphincterotome methods failed to achieve deep bile duct cannulation, if the pancreatic duct was cannulated, whether to perform double guidewire method or cannulation after the insertion of a pancreatic duct stent depended on the individual endoscopist. All the above-mentioned methods were regarded as conventional cannulation methods. Difficult biliary cannulation was defined when the conventional cannulation methods failed to achieve deep bile duct cannulation. Although there was no strict definition of difficult biliary cannulation in terms of cannulation frequency, the cannulation time for difficult cases usually exceeded 20 min before 2015. After 2015, the early NKPS strategy was usually adopted^24, 25^. Early NKPS was performed when cannulation time more than 5 min or unintentional pancreatic duct cannulation more than once^25^.

### Procedures of NKPS

For patients with difficult biliary cannulation, NKPS was performed immediately after the failure of the conventional cannulation methods during the same endoscopic session. The needle-knife sphincterotome (Rx Needle-Knife XL; Boston Scientific Corporation, Marlborough, USA) and an ICC 200 or VIO 200D electrosurgical unit (ERBE, Tübingen, Germany), which produced blended current, were used for NKPS. After making a puncture in the papilla above the orifice, the incision was made upward along the axis of the bile duct from the papillary orifice. The cut was extended until the common bile duct (CBD) was exposed, followed by a small incision in the biliary sphincter muscle. The CBD was then cannulated directly with the closed needle-knife or with a wire-guided cannula/sphincterotome. Failure of the initial NKPS procedure was defined as failure to expose the CBD or failure to place a catheter/sphincterotome deeply into the CBD to obtain satisfactory cholangiography.

### Patients and study design

During the 15 years from January 2004 to December 2018, we consecutively enrolled patients who underwent NKPS due to difficult biliary cannulation in our institution. The patients’ data were retrospectively retrieved from the database of our Therapeutic Endoscopy Center. During the study period, five endoscopists (A–E) performed 512 NKPS procedures for patients with difficult biliary cannulation. This study was reviewed and approved by the Ethics Committee of the Chang Gung Memorial Hospital (IRB No.: 202100304B0). Since this is a retrospective study using clinical routine treatment or diagnostic medical records, and no human immunodeficiency virus-positive cases were involved, the Chang Gung Medical Foundation Institutional Review Board approved the waiver of the participant's consent. All methods were carried out in accordance with relevant guidelines and regulations.

**Figure 1:**
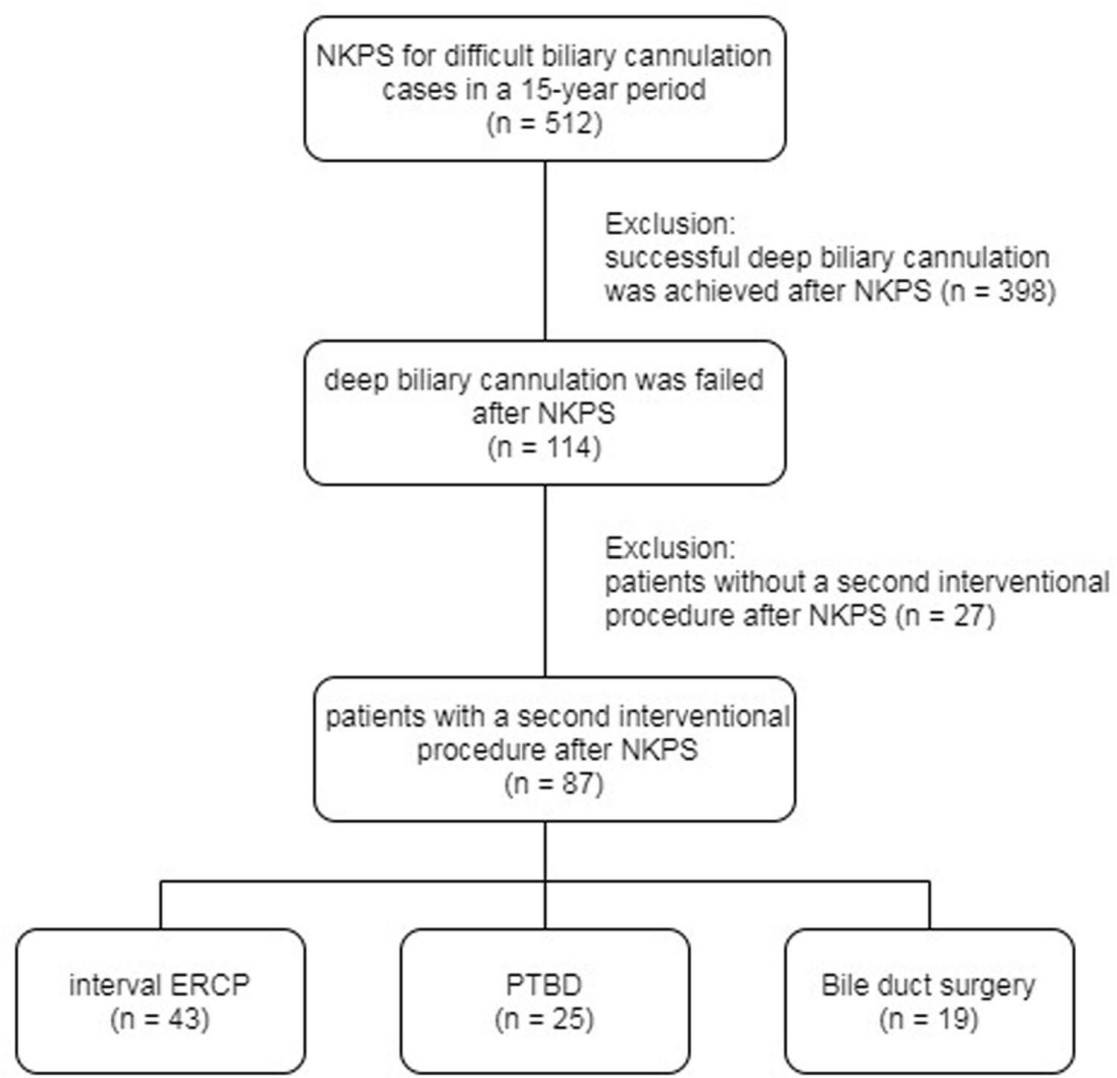
Flow chart of the study**.** *NKPS* needle knife precut sphincterotomy, *ERCP* endoscopic retrograde cholangiography, *PTBD* percutaneous transhepatic biliary drainage.

The exclusion criteria were (1) patients with successful deep bile duct cannulation by the NKPS (n = 398 or 77.7%); (2) patients with initial NKPS failure but not receiving a second interventional procedure, n = 27). Therefore, a total of 87 patients who met the inclusion and exclusion criteria were enrolled in this study (Fig. 1). Among these 87 patients with a second interventional procedure, 31 patients (35.6%) received interval ERCP, 12 patients (13.8%) received interval ERCP via PTE-RVs, 21 patients (24.1%) received PTCD, 4 patients (4.36%) received percutaneous transhepatic gallbladder drainage (PTGBD), and 19 patients (21.8%) received BDS. What kind of second interventional procedure was performed depending on the decision of the attending physician in the ward. For statistical analysis, patients who received interval ERCP and interval ERCP via PTE-RVs were combined as interval endoscopic treatment group (IET group, n = 43); patients who received PTCD and PTGBD were combined as percutaneous transhepatic biliary drainage group (PTBD group, n = 25), and patients who received BDS were classified as BDS group.

In the IET group, in case of the first ERCP failure, we tended to reserve the second ERCP to the most experienced endoscopist (71% of procedures were performed by endoscopist A). In the second ERCP, conventional cannulation methods were performed when the bile duct had been exposed (especially by bile staining or bile flow identification). If the conventional cannulation methods failed to achieve deep bile duct cannulation under the above circumstance, or if the bile duct could not be found on the second ERCP, a second pre-cut was performed.

The comparisons between groups were divided into three parts. The first part was the patients’ baseline characteristics, including demographic data, liver biochemistry tests, and the major papilla morphology. Demographic data included age and gender. Liver biochemistry tests included serum levels of aspartate aminotransferase (AST), alanine aminotransferase (ALT), alkaline phosphatase (Alk-P), and total bilirubin. The major papilla morphology included periampullary diverticulum and surgically altered anatomy. The second part was the factors that might affect the choice of endoscopic treatment or non-endoscopic treatment for the second interventional procedure. These factors included indications and adverse events of the first ERCP procedures, and inpatient departments. The indications for the first ERCP were choledocholithiasis, malignant biliary stricture, and benign biliary stricture or leakage. The adverse events of the first ERCP included bleeding and hemostasis during the NKPS, delayed post-ERCP bleeding, and post-ERCP pancreatitis. Bleeding and hemostasis during the NKPS referred to bleeding caused by precut, and hemostasis was to prevent bleeding from blocking the endoscopic view. The criteria of delayed post-ERCP bleeding and post-ERCP pancreatitis were defined according to Cotton et al.^26^. Inpatient departments included internal medicine and surgery. The third part was the comparison of the results of the second interventional procedures, including time interval between the first and second interventional procedures, technical success rate, early adverse events, length of hospital stay, and 30-day mortality.

### Statistical analysis

Since the aim of the study was to report the outcomes between endoscopic and non-endoscopic treatments, comparisons were made between the IET group and the PTBD group and between the IET group and the BDS group. Continuous variables were shown as median with range, and statistical analysis for continuous variables was performed with the Mann–Whitney U test. For categorical variables, the chi-square test or Fisher exact test was used for statistical analysis whenever appropriate. Logistic regression analysis was performed to identify predictors associated with the second ERCP cannulation failure in the IET group. Statistical analyses were performed using SPSS software (version 20.0; SPSS, Inc., Chicago, IL). A two-tailed p-value of < 0.05 was considered statistically significant.

## Results

There were 43 (49.4%), 25 (28.7%), and 19 (21.8%) patients in the IET, PTBD, and BDS groups, respectively. The patients’ baseline characteristics are demonstrated in Table 1. Compared with the PTBD group, patients in the IET group had significantly lower serum levels of Alk-P (median, 127 U/L vs. 211 U/L, p = 0.028) and total bilirubin (median, 2.6 mg/dL vs. 9.5 mg/dL, p = 0.007). Furthermore, the periampullary diverticulum was found to be significantly more prevalent in the IET group (41.9% vs. 8.0%, p = 0.003). Compared with the BDS group, the only significant finding was that patients in the IET group were older (median, 75 years vs. 60 years, p = 0.004).

**Table 1:** Clinical, laboratory, and endoscopic characteristics of the patients.

Variables	IET group (n = 43)	PTBD group (n = 25)	BDS group (n = 19)	p-value
Median age, year (range)	75 (31–93)^#^	72 (45–88)	60 (27–79)^#^	0.004^#^
Gender (male), n (%)	21 (48.8%)	13 (52.0%)	12 (63.2%)	NS
**Liver biochemistry before 1st ERCP**				
AST, median (range) (U/L)	70 (13–371)	79 (20–349)	132 (19–437)	NS
ALT, median (range) (U/L)	84 (13–447)	108 (11–286)	199 (40–509)	NS
Alkaline-P, median (range) (U/L)	127 (36–1281)*	211 (59–952)*	152.5 (57–869)	0.028*
Total Bilirubin, median (range) (mg/dL)	2.6 (0.4–9.2)*	9.5 (0.8–16.0)*	5.6 (0.9–8.6)	0.007*
**Morphology of the major papilla**				
Periampullary diverticulum, n	18 (41.9%)*	2 (8.0%)*	4 (21.1%)	0.003*
Surgically altered anatomy, n	3 (7.0%)	4 (16.0%)	2 (10.5%)	NS

*IET* interval endoscopic treatment, *PTBD* percutaneous transhepatic biliary drainage, *BDS* bile duct surgery, *AST* aspartate aminotransferase, *ALT* alanine aminotransferase, *NS* not significant between the IET group and the PTBD group and between the IET group and the BDS group.

*Statistical significance between the IET group and the PTBD group.

^#^Statistical significance between the IET group and the BDS group.

Table 2 lists the factors that might affect the choice of endoscopic versus non-endoscopic treatment for the second interventional procedure. Indication of first ERCP included choledocholithiasis (n = 50), malignant biliary stricture (n = 30), and benign biliary stricture or leakage (n = 7). Among the 30 patients with malignant biliary stricture, 26 cases were distal bile duct stricture and 4 cases were hilar obstruction. Compared with the PTBD group, the incidence of choledocholithiasis in the IET group was significantly higher (69.8% vs. 16.0%, p < 0.001), and the incidence of malignant biliary stricture was lower (20% vs. 77.3%, p < 0.001). There was no significant difference in any ERCP indication between the IET group and the BDS group. However, most of the indications for ERCP in the BDS group were choledocholithiasis (16/19 or 84.2%). Compared with the BDS group, there were significantly fewer patients referred from the surgical department to the IET group (46.5% vs. 89.5%, p = 0.001). In fact, of the 19 patients who received BDS as the second interventional procedure, 17 (89.5%) came from the surgical department, while only 2 (10.5%) came from the medical department. Regarding ERCP complications, there were no significant differences between the IET and PTBD groups and between the IET and BDS groups.

**Table 2:** The factors that might affect the choice of endoscopic versus non-endoscopic therapy for the second interventional procedure.

Variables	IET group (n = 43)	PTBD group (n = 25)	BDS group (n = 19)	p-value
**Indications of the first ERCP**				
Choledocholithiasis	30 (69.8%)*	4 (16.0%)*	16 (84.2%)	< 0.001*
Malignant biliary stricture	9 (20.9%)*	19 (76.0%)*	2 (10.5%)	< 0.001*
Distal bile duct obstruction, n	7 (77.8%)	17 (89.5%)	2 (100%)	–
Hilar obstruction, n	2 (22.2%)	2 (10.5%)	0	–
Benign biliary stricture or leak	4 (9.3%)	2 (8.0%)	1 (5.3%)	NS
**Adverse events of the first ERCP, n**				
bleeding and hemostasis during NKPS^†^	19 (44.2%)	9 (36%)	6 (31.6%)	NS
Delayed Post-ERCP bleeding	1 (2.3%)	0	0	NS
pancreatitis	2 (4.7%)	0	2 (10.5%)	NS
Inpatient department				
Medical department	23 (53.5%)^#^	8 (32.0%)	2 (10.5%)^#^	0.001^#^
Surgical department	20 (46.5%)^#^	17 (68.0%)	17 (89.5%)^#^	0.001^#^

*IET* interval endoscopic treatment, *PTBD* percutaneous transhepatic biliary drainage, *BDS* bile duct surgery, *NS* not significant between the IET group and the PTBD group and between the IET group and the BDS group.

^†^Bleeding caused by precut, and hemostasis to prevent bleeding from blocking the endoscopic view.

*Statistical significance between the IET group and the PTBD group.

^#^Statistical significance between the IET group and the BDS group.

The outcomes of the second interventional treatment are listed in Table 3. In the IET group, 15 patients (34.9%) needed a second NKPS during the interval ERCP. Compared with the PTBD group, the IET group had a significantly longer time interval between the first and second interventional procedures (median, 4 days vs. 2 days, p = 0.001), lower technique success rate (34/43 or 79.1% vs. 100%, p = 0.021), and shorter length of hospital stay after the second procedure (median, 7 days vs. 18 days, p < 0.001). Among the 9 patients in the IET group with failed second ERCP, two patients received a third ERCP, one patient received PTBD, five patients received BDS, and one patient did not receive a third interventional procedure. The factors associated with the cannulation failure of the second ERCP in the 31 patients who did not undergo rendezvous procedures were further evaluated (Table 4). However, there were no predictors determined in univariate and multivariate analysis. Compared with the BDS group, there were no significant differences in any outcome parameters in the IET group. The rate of complications related to the second interventional procedure was 7% (3/43) in the IET group, 8% (2/25) in the PTBD group, and 15.8% (3/19) in the BDS group. Although there was no statistical difference in the rate of complications, the types of complications in each group were quite different. The types of complications in the IET group included 1 case of precut bleeding and 2 cases of biliary tract infection. In the PTBD group, there was 1 case of biliary tract infection and 1 case of acute biliary perforation with bile peritonitis. In the BDS group, there was 1 case of hemobilia, 1 case of hemoperitoneum, and 1 case of bile duct perforation. The 30-day mortality rate in IET, PTBD and BDS groups was 7% (3/43), 16% (4/25) and 0%, respectively (p = not significant). The causes of death of the 3 patients in the IET group were new onset of ischemic stroke, decompensated liver cirrhosis, and advanced hepatocellular carcinoma. Among the 4 patients in the PTBD group, the causes of death were advanced Klatskin tumor, advanced lung cancer, frequent seizure attacks, and decompensated liver cirrhosis combined with hepatocellular carcinoma. In each group, none with 30-day mortality was caused by the second interventional procedure.

**Table 3:** Outcome comparisons between endoscopic versus non-endoscopic treatment.

Variables	IET group (n = 43)	PTBD group (n = 25)	BDS group (n = 19)	p-value
Days between 1st and 2nd procedures, median (range)	4 (1–20)*	2 (0–36)*	3 (1–11)	0.001*
Technical success, n	34 (79.1%)*	25 (100%)*	18 (94.7%)	0.021*
Early complications related to the second treatment, n	3 (7.0%)	2 (8.0%)	3 (15.8%)	NS
Length of hospital stay after the second treatment, median days (range)	7 (2–40)*	18 (2–55)*	8 (2–37)	< 0.001*
30-day mortality, any causes	3 (7.0%)	4 (16.0%)	0	NS
30-day mortality related to the second treatment	0	0	0	NS

*IET* interval endoscopic treatment, *PTBD* percutaneous transhepatic biliary drainage, *BDS* bile duct surgery, *NS* not significant between the IET group and the PTBD group and between the IET group and the BDS group.

*Statistical significance between the IET group and the PTBD group.

^#^Statistical significance between the IET group and the BDS group.

**Table 4:** Univariate and multivariate analysis of factors associated with the second ERCP failure of patients in the interval endoscopic treatment group who did not undergo rendezvous procedures.

Variables	Univariate analysis		Multivariate analysis	
	OR (95% CI)	p-value	OR (95% CI)	p-value
**Age**				
< 70 years	1			
≥ 70 years	1.156 (0.241–5.530)	0.856		
**Sex**				
Male	1			
Female	1.156 (0.241–5.530)	0.856		
**Indications of the first ERCP**				
Non-malignant	1			
Malignant	1.778 (0.171–18.534)	0.630		
**Diverticulum**				
No	1			
Yes	1.143 (0.223–5.866)	0.873		
**Adverse events of the first ERCP**				
No	1			
Yes	2.625 (0.146–47.183)	0.513		
**Interval between two ERCP**				
< 4 days	1		1	
≥ 4 days	0.419 (0.082–2.106)	0.290	0.444 (0.085–2.325)	0.337
**Need precut on the second ERCP**				
No	1		1	
Yes	2.679 (0.545–13.157)	0.225	2.531 (0.502–12.772)	0.261

*ERCP* endoscopic retrograde cholangiography, *OR* odds ration.

## Discussion

ERCP, PTBD, and BDS are alternative interventions used in the management of biliary diseases. ERCP has become the preferred treatment for most biliary diseases, however, it is not always successful even with NKPS even in high-volume medical centers^3^. There is no consensus or guidelines on the management of these patients with initial NKPS failure due to difficult biliary cannulation. Several studies have shown that interval ERCP may be a viable treatment option after the initial NKPS failure, with a success rate of 68%–78%^17, 18, 27–29^. Our success rate was 79.1% (34/43), which added to the evidence for the feasibility of interval ERCP. However, 12 of our 43 patients received interval ERCP via PTE-RVs. The success rate of interval ERCP with PTE-RVs was significantly higher than that of interval ERCP without PTE-RVs (12/12 or 100% vs. 22/31 or 71%, p = 0.04). To our knowledge, no study compares the results between interval ERCP with and without PTE-RVs. Besides, PTE-RV appears to be preferable to PTBD because PTE-RV allows physicians to perform transhepatic punctures using only small-caliber catheters, thereby reducing complications^19^. Therefore, PTE-RVs can be used to achieve biliary access when the standard methods for biliary cannulation fail^25^.

In theory, hyperemia and edema of the papilla caused by NKPS will improve over time, which may increase the success rate of the second ERCP. However, the optimal interval between the first and second ERCP has not yet been determined. Kim J. et al. reported that the success rate after one day was significantly lower than that 2–3 days later (65.7% vs. 88.2%, P = 0.027)^27^. Another study conducted by Colan-Hernandez et al. concluded that a second ERCP should be delayed by at least 4 days because the procedure within 4 days after the initial precut was the only significant factor associated with the second ERCP failure^17^. However, we were unable to determine any factors related to the failure of the second ERCP, including the time interval.

The use of PTBD as an initial treatment for biliary diseases has declined in recent decades because PTBD is associated with adverse events accounting for 9%–13% and serious adverse events accounting for 4%–8%^3, 23^. Besides, PTBD reduces the patient's quality of life due to external drainage, and the drainage often needs to be replaced. Furthermore, it is difficult to carry out when the intrahepatic bile ducts (IHDs) are not dilated. Nevertheless, endoscopic biliary drainage is considered better than PTBD in patients with coagulopathy or ascites^21^. However, as shown in this study, PTBD is still a useful rescue therapy in which initial ERCP fails, especially for resectable malignant biliary obstruction^30, 31^.

In a recent US nationwide longitudinal study, ERCP has almost completely replaced the BDS to treat choledocholithiasis, which may be due to the improvement of the therapeutic capacity and safety of ERCP^23^. However, due to lack of study, it is unclear whether patients who fail the initial ERCP will still experience this trend. No trend toward ERCP was found in the present study because the inpatient department seemed to affect the decision-making on the endoscopic or surgical treatment. Among the patients receiving BDS, 84.2% of patients had choledocholithiasis, and all were from the surgical department. In contrast, none of the patients with choledocholithiasis in the medical department received BDS. Just like the treatment of colon polyps, this is an interesting phenomenon because physician expertise is often closely related to treatment strategy^32^. However, compared with the IET group, the BDS group had a higher incidence of procedure-related complications (15.8% vs. 7%, although not statistically significant) and more serious. As mentioned above, IET has an acceptably high success rate. Therefore, it is reasonable to provide endoscopic rather than surgical treatment for these patients with choledocholithiasis.

The major limitation of this study is its retrospective design, therefore, the patients in each group were not randomized. Most patients in the PTBD group were patients with malignant biliary obstruction (hence, dilated IHD). This selection bias might be the reason for the higher technical success rate of the PTBD group than the IET group. However, due to the low initial failure rate of NKPS in an experienced center, it was difficult to conduct prospective randomized studies. Second, endoscopic ultrasound-guided biliary drainage (EUS-BD) is increasingly used in patients who fail standard ERCP. EUS-BD may be preferred over PTBD because of better clinical success, fewer post-procedure adverse events, and a lower rate of re-intervention^33^. However, due to a lack of adequate expertise, it was not available in our institution during the study period.

In conclusion, IET has an acceptable success rate and less severe complications and should be tried after initial failed NKPS before contemplating more invasive interventions such as BDS. PTBD may be an alternative rescue therapy for patients with malignant biliary obstruction.
